# Dialyzer surface area is a significant predictor of mortality in patients on hemodialysis: a 3-year nationwide cohort study

**DOI:** 10.1038/s41598-021-99834-4

**Published:** 2021-10-18

**Authors:** Masanori Abe, Ikuto Masakane, Atsushi Wada, Shigeru Nakai, Kosaku Nitta, Hidetomo Nakamoto

**Affiliations:** 1 The Committee of Renal Data Registry, Japanese Society for Dialysis Therapy, Tokyo, Japan; 2 Division of Nephrology, Hypertension and Endocrinology, Department of Internal Medicine, Nihon University School of Medicine, 30-1 Oyaguchi, Kami-cho, Itabashi-ku, Tokyo, 173-8610 Japan; 3 Yabuki Hospital, Yamagata, Japan; 4 Department of Nephrology, Kitasaito Hospital, Asahikawa, Japan; 5 Department of Clinical Engineering, Fujita Health University, Toyoake, Aichi Japan; 6 Department of Nephrology, Tokyo Women’s Medical University, Tokyo, Japan; 7 Department of General Internal Medicine, Saitama Medical University, Saitama, Japan

**Keywords:** Nephrology, Renal replacement therapy, Haemodialysis

## Abstract

A target Kt/V of > 1.4 and use of a high-flux dialyzer are recommended for patients on hemodialysis. However, there is little information on the relationship between the dialyzer surface area and mortality in these patients. In this nationwide cohort study, we aimed to clarify this relationship by analyzing data from the Japanese Society for Dialysis Therapy for 2010–2013. We enrolled 234,638 patients on hemodialysis who were divided according to quartile for dialyzer surface area into the S group (small, < 1.5 m^2^), M group (medium, 1.5 m^2^), L group (large, 1.6 to < 2.0 m^2^), or XL group (extra-large, ≥ 2.0 m^2^). We assessed the association of each group with 3-year mortality using Cox proportional hazards models and performed propensity score matching analysis. By the end of 2013, a total of 53,836 patients on dialysis (22.9%) had died. There was a significant decrease in mortality with larger dialyzer surface areas. The hazard ratio (95% confidence interval) was significantly higher in the S group (1.15 [1.12–1.19], *P* < 0.0001) and significantly lower in the L group (0.89 [0.87–0.92] *P* < 0.0001) and XL group (0.75 [0.72–0.78], *P* < 0.0001) than in the M group as a reference after adjustment for all confounders. Findings were robust in several sensitivity analyses. Furthermore, the findings remained significant after propensity score matching. Hemodialysis using dialyzers, especially super high-flux dialyzers with a larger surface area might reduce mortality rates, and a surface area of ≥ 2.0 m^2^ is superior, even with the same Kt/V.

## Introduction

Patients with end-stage kidney disease on dialysis have increased morbidity and mortality rates because of uremic toxin accumulation. Uremic toxins are classified according to their molecular weight as small (< 500 Da, e.g., urea), middle (> 500 Da, e.g., β_2_-microglobulin [β2MG]), or protein-bound^1,2^. Hemodialysis remains the main modality of renal replacement therapy in patients with end-stage kidney disease, and one of its purposes is to achieve adequate uremic toxin removal. Therefore, several guidelines recommend the Kt/V value for measurement of the dialysis dose delivered^3–5^. Also, given that a higher serum β2MG level has been found to predict all-cause mortality independently of several confounding factors^6^, European Best Practice Guidelines recommend β2MG as a marker for middle-molecular-weight uremic toxins and stress the need for their removal in patients on hemodialysis^7^. The Japanese Society for Dialysis Therapy (JSDT) guidelines also recommend regular monitoring of the serum β2MG level and that the maximum pre-hemodialysis level should be < 30 mg/L^3^.

To increase uremic toxin removal, the Renal Association recommends use of high-flux membrane dialyzers and a minimum dialysis time of 12 h per week for patients treated three times weekly^5^. Furthermore, the Kidney Disease Outcomes Quality Initiative (KDOQI) and JSDT guidelines recommended a dialysis dose assessed by single-pool Kt/V for urea (Kt/V) of 1.4 per hemodialysis session and a minimum delivered Kt/V of 1.2^3,4^. Kt/V can be increased by increasing the blood or dialysate flow rate, the dialyzer surface area, and the treatment time. Although treatment time and membrane flux determine the Kt/V, prolonged hemodialysis treatment time was found to be associated with lower mortality risk even with the same Kt/V level^8^. Therefore, predictors of prognosis other than Kt/V might exist for hemodialysis patients. The associations of mortality with Kt/V, blood flow rate, and treatment time have often been discussed, but the relationship between dialyzer surface area and mortality has not been investigated to date. In Japan, super high-flux or high-performance membrane (HPM) dialyzers have been used since 2005. HPM dialyzers are defined as having high hydraulic permeability, high solute permeability, particularly for middle-molecular-weight substances and uremic toxins with molecular weights of 10–30 kDa, high biocompatibility, and β2MG clearance > 50 mL/min^3,9,10^. Therefore, increasing the use of super high-flux dialyzers with a greater surface area might contribute to increased removal of uremic toxins and better prognosis in patients on hemodialysis. Using dialyzers with a greater surface area may also help to lower the mortality risk irrespective of the Kt/V level.

The aim of this large registry study was to investigate the impact of dialyzer surface area on the clinical outcome of patients undergoing hemodialysis in Japan.

## Methods

### Data source and study design

All data analyzed in this study were obtained from the JSDT Renal Data Registry (JRDR) and were collected by a questionnaire-based nationwide survey, the design and methods of which have been reported elsewhere^11,12^. The study had a right-censoring prospective cohort design and analyzed JRDR data collected from December 31, 2010 (baseline)^13^ to December 31, 2013^14^. Eligibility criteria were as follows: age ≥ 20 years; undergoing maintenance dialysis in Japan at the end of 2010; and 3 years of follow-up from 2010 to 2013. Exclusion criteria were dialysis < 3 times weekly or for < 2 h daily, organ transplantation, peritoneal dialysis, and missing data for date of birth, time of initiation of dialysis, dialyzer surface area, or outcome. Patients were divided into quartiles according to dialyzer surface area: an S group (small, < 1.5 m^2^), an M group (medium, 1.5 m^2^), an L group (large, 1.6 to < 2.0 m^2^), and an XL group (extra-large, ≥ 2.0 m^2^). The M group was defined as the reference group because dialyzers with a surface area of 1.5 m^2^ are the most widely used in Japan.

### Covariate and outcome data

Baseline patient and laboratory data collected from the JRDR database in 2010 included age, sex, duration and modality of dialysis, body mass index (BMI; calculated as post-hemodialysis body weight [kg]/height [m] squared), cause of end-stage kidney disease, laboratory measures including pre-hemodialysis hemoglobin, serum albumin, calcium, phosphate, intact parathyroid hormone, β2MG, and C-reactive protein (CRP) levels, Kt/V, hemodialysis time, normalized protein catabolic rate (nPCR), and history of myocardial infarction, cerebral hemorrhage, cerebral infarction, or limb amputation. Ultrafiltration rate was defined as the rate of volume removal at hemodialysis (mL/h/kg bodyweight) and was based on the weight change per treatment time, using the post-hemodialysis weight as the denominator. Kt/V and nPCR were calculated using Shinzato’s formula^15^. A simplified creatinine index (SCI) was calculated using the Canaud formula^16^. Equations for calculating Kt/V, nPCR, and SCI are shown in Supplementary Table 1. Dialyzers were classified as low, medium, high, or super high flux based on β2MG clearance and the ultrafiltration rate. The definitions and classifications of dialyzer flux type are shown in Supplementary Table 2. A super high-flux dialyzer is defined as having a β2MG clearance ≥ 50 mL/min and an ultrafiltration rate of 50 mL/h/mmHg. We defined reference ranges for the laboratory data such that patients with measured values outside of the following ranges were considered outliers and were excluded from the analysis: height 120–200 cm, body weight 20–150 kg, serum albumin 1.0–5.0 g/ dL, CRP < 30 mg/dL, hemoglobin 5.0–20.0 g/dL, and i-PTH < 3000 pg/mL.

The main outcome measures were time to all-cause mortality, cardiovascular (CV) mortality, and non-CV mortality during the 3-year observation period. Follow-up ended at the time of death, withdrawal, kidney transplantation, or December 31, 2013, whichever occurred first. CV mortality was defined as death caused by heart failure, acute myocardial infarction, arrhythmia, valvular disease, subarachnoid hemorrhage, cerebral hemorrhage, or cerebral infarction or as sudden death. Non-CV mortality was defined as death from a non-CV cause, including infectious disease and malignancy.

### Statistical methods

The data are summarized as proportions with mean ± standard deviation (SD) or median [interquartile range] as appropriate. The chi-square test was used to analyze categorical variables. Student’s *t*-test was used to analyze continuous variables. Categorical data were compared between groups using repeated-measures analysis of variance and Tukey’s honestly significant difference test or the Kruskal–Wallis test, as appropriate. Missing covariate data were imputed by the mean or median of the existing values, whichever was most appropriate.

### Analysis of predictors of mortality from baseline demographic and laboratory data

To evaluate potential predictors of mortality, univariate Cox proportional hazards regression analysis was used to examine whether basic factors at baseline (e.g., age, sex, cause of end-stage kidney disease, CV comorbidity, and duration of dialysis) predicted survival for up to 3 years of follow-up. To examine the relationship between category of hemodialysis duration and risk of death, we divided patients into five a priori categories based on duration of hemodialysis (< 2, 2 to < 5, 5 to < 10, 10 to < 20, and ≥ 20 years). We also categorized patients by dialysis-related factors, including Kt/V, β2MG level, ultrafiltration rate, and hemodialysis time. To examine the dose–response association between the Kt/V categories and risk of death, we divided patients into six a priori categories based on Kt/V (< 1.0 and ≥ 1.8, with intervening increments of 0.2). BMI, hemoglobin, serum albumin, nPCR, SCI, and CRP levels were included as nutritional- and inflammation-related factors. To examine the dose–response associations of serum albumin level and nPCR with risk of death, we divided patients into five a priori categories based on serum albumin levels (< 3.0 and ≥ 4.5, with intervening increments of 0.5) and on nPCR (< 0.6 and ≥ 1.2, with intervening increments of 0.2). Age, hemodialysis time, ultrafiltration rate, β2MG, BMI, hemoglobin, SCI, and CRP levels were analyzed as continuous variables.

### Outcome analysis according to dialyzer surface area

To examine all-cause mortality, we performed unadjusted and adjusted analyses that included all predictors found to be significant on univariate Cox proportional hazards regression analysis. Survival according to dialyzer surface area was estimated using the Kaplan–Meier method and compared using the log-rank test. Survival analyses with multivariate Cox proportional hazards regression analysis were used to examine whether basic factors at baseline (e.g., age, sex, hemodialysis duration, and CV comorbidity) predicted survival for up to 3 years of follow-up. Additional analyses were performed with adjustment for both basic factors and dialysis-related factors, Kt/V, β2MG level, ultrafiltration rate, and hemodialysis time. Further analyses were then performed with adjustments for basic factors, dialysis-related factors, and nutrition-related and inflammation-related factors (e.g., BMI and levels of hemoglobin, albumin, and CRP). Associations were examined between all-cause mortality, CV mortality, and non-CV mortality according to dialyzer surface area. The M group was defined as the reference group because M dialyzers are the most widely used. The validity of the proportional hazards assumption was examined graphically and by formal statistical testing. Multicollinearity was examined with the variance inflation factor (VIF), and covariates of VIF < 5 were used in the final adjusted Cox proportional hazards regression analysis.

To assess the robustness of the main results, several sensitivity analyses were performed. First, an age-stratified subgroup analysis was conducted by age < 68 and ≥ 68 years (the median value). Second, a subgroup analysis was performed based on history of cardiovascular disease (CVD) and diabetes mellitus (DM) status, given that dialyzers with a large surface area are unlikely to be used in patients with impaired cardiac function and the higher rate of comorbid CVD in patients with DM. Third, a subgroup analysis was conducted by BMI < 21 and ≥ 21 (the median value). Fourth, a stratified analysis was conducted according to serum β2MG and albumin levels. Fifth, analysis was conducted separately for the four flux categories of dialyzer (low, medium, high, and super high). Finally, considering that dialyzer surface area might be associated with Kt/V, a subgroup analysis was performed according to Kt/V quartile. We also examined whether the association between dialyzer surface area and mortality differed according to Kt/V level, by creating multiplicative interaction terms between Kt/V and dialyzer surface area and used the Wald test to assess interactions.

Significant differences in covariates at baseline were adjusted for by propensity score matching. Covariates for calculating propensity scores were obtained prior to starting hemodialysis. To calculate the propensity score for each patient, we performed multivariable logistic regression analysis using dialyzer surface area as the dependent variable and the significant predictors as independent variables, followed by logit transformation. Propensity scores were derived for age, sex, duration of dialysis, comorbid CVD, DM status, Kt/V, β2MG, dialyzer type, BMI, nPCR, SCI, and serum albumin, hemoglobin, phosphate, calcium, intact parathyroid hormone, and CRP levels. The propensity scores were calculated to a significance of 14 decimal points. Patients in the M (reference) group were matched in a 1:1 ratio with the other groups. Matching of patients in 2 groups at a 1:1 ratio was performed using nearest available matching with a caliper width of 0.2 × SD, where SD is the SD of logit values of all patients in each group. All-cause mortality was compared in propensity score-matched patients.

All analyses were performed using JMP® version 13.0 (SAS Institute Inc., Cary, NC). A P-value less than 0.05 was considered statistically significant.

### Ethical approval

The study protocol was approved by the JSDT Medicine Ethics Committee and conducted according to the principles of the Declaration of Helsinki, Japanese privacy protection laws, and the 2015 Ethical Guidelines for Medical and Health Research Involving Human Subjects published by the Ministry of Education, Science, and Culture and the Ministry of Health, Labour and Welfare. Our analyses used existing data without any individual patient identifiers, and the need for informed consent was waived in view of the anonymity of the data. This study is registered with the University Hospital Medical Information Network (UMIN000018641).

## Results

**Figure 1:**
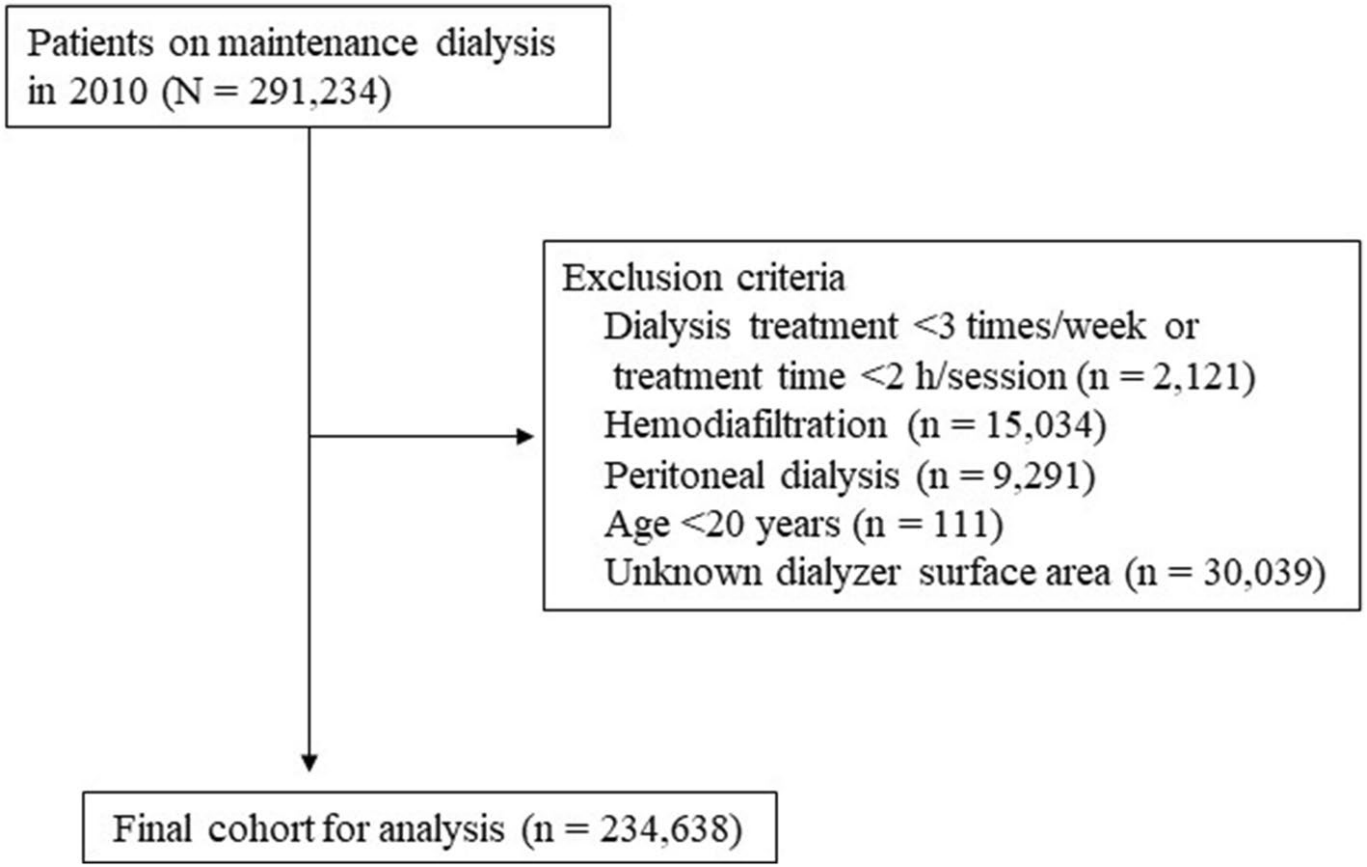
Flowchart showing the process used to extract the data analyzed in this study.

Figure 1 outlines the data extraction process from an original data set of 291,234 patients at the end of 2010, from which 234,638 patients remained for analysis after exclusions. Table 1 shows the baseline characteristics of these 234,638 patients (age, 65.5 ± 12.4 years; male, 62.4%; median duration of dialysis, 6 years) with data on dialyzer surface area and also the 30,039 patients without data on dialyzer surface area. The underlying condition was chronic glomerulonephritis in 38.2%, diabetic nephropathy in 36.5%, nephrosclerosis in 8.5%, polycystic kidney disease in 3.4%, and other or unknown in 13.4%. In total, 53,836 deaths (22.9%) were recorded during the observation period, comprising 23,446 CV-related deaths, 10,755 infection-related deaths, 5243 cancer-related deaths, and 14,392 other deaths.

**Table 1:** Demographics, clinical characteristics, and laboratory findings in a 3-year cohort of 264,677 patients on hemodialysis, including 234,638 patients with data on dialyzer surface area.

Variable	Dialyzer surface area data available	Dialyzer surface area data unavailable	*P* value
Patients, n (% female)	234,638 (37.6)	30,039 (37.1)	0.113
Age (years)	65.5 ± 12.4	65.9 ± 12.5	< 0.0001
Duration of dialysis (years)	6 [3–11]	5 [2–10]	< 0.0001
**Primary kidney disease (%)**			< 0.0001
Glomerulonephritis	38.2	38.0	
Diabetic nephropathy	36.5	36.6	
Nephrosclerosis	8.5	8.2	
Polycystic kidney disease	3.4	3.0	
Other	13.4	14.2	
**Comorbid CVD (%)**	24.5	31.4	< 0.0001
Coronary artery disease	8.2	13.4	
Ischemic stroke	15.8	21.7	
Hemorrhagic stroke	5.2	7.2	
Limb amputation	3.1	5.6	
Body mass index	21.3 ± 3.7	21.2 ± 3.7	0.011
β_2_-microglobulin (mg/L)	26.7 ± 7.1	25.7 ± 7.6	< 0.0001
Kt/V	1.40 ± 0.31	1.41 ± 0.30	0.0029
Dialyzer surface area (m^2^)	1.6 [1.5–1.9]	–	–
**Type of dialyzer**			< 0.0001
Low-flux	0.9	0.8	
Medium-flux	1.0	1.3	
High-flux	3.9	7.2	
Super high-flux	94.2	90.7	
Hemoglobin (g/dL)	10.5 ± 1.3	10.5 ± 1.4	< 0.0001
Calcium (mg/dL)	8.9 ± 0.8	8.9 ± 0.8	0.0002
Phosphate (mg/dL)	5.2 ± 1.5	5.2 ± 1.4	0.049
Intact PTH (pg/mL)	116 [58–197]	126 [67–207]	0.297
C-reactive protein (mg/dL)	0.1 [0.1–0.4]	0.1 [0.1–0.5]	< 0.0001
Albumin (g/dL)	3.7 ± 0.4	3.6 ± 0.5	< 0.0001
nPCR (g/kg/day)	0.87 ± 0.18	0.86 ± 0.17	< 0.0001
SCI (mg/kg/day)	93.5 ± 24.2	92.1 ± 23.8	< 0.0001

Data are expressed as mean ± standard deviation (SD), number (%), or median [interquartile range].

*CVD* cardiovascular disease, *nPCR* normalized protein catabolic rate, *PTH* parathyroid hormone, *SCI* simplified creatinine index.

### Predictors of all-cause mortality in 234,638 patients on hemodialysis

Supplementary Table 3 shows the HRs and 95% CIs for variables that were evaluated as potential predictors of mortality. Significant basic predictors were male sex, older age, longer duration of dialysis, comorbid CVD, and presence of DM. In terms of dialysis-related factors, a higher Kt/V, a lower β2MG level, and longer dialysis time was associated with a lower risk of mortality. For nutrition-related and inflammation-related factors, poor nutritional status, indicated by lower hemoglobin, serum albumin, BMI, nPCR, and SCI values, was associated with a higher mortality risk, as was a higher inflammatory status (indicated by a higher CRP level).

### Clinical and demographic characteristics according to dialyzer surface area

Table 2 shows the patient demographics and characteristics according to the dialyzer surface area. Patients treated with a small dialyzer surface area were older, more likely to be female, and had higher rates of comorbid CVD and lower BMI, serum albumin, nPCR, and SCI levels. In contrast, patients treated using a larger dialyzer surface area were younger, more likely to be male, had lower rates of comorbid CVD and DM, and had higher BMI, β2MG, nPCR, and SCI levels.

**Table 2:** Demographic, clinical, and laboratory data for 234,638 patients on hemodialysis according to dialyzer surface area.

Variable	S group	M group	L group	XL group	*P* value
n (%)	51,174 (21.8)	62,748 (26.7)	63,714 (27.2)	57,002 (24.3)	
Age, years	72.9 ± 11.3	68.4 ± 11.5	65.4 ± 11.6	59.7 ± 11.7	< 0.0001
Sex, female, %	58.6	44.5	33.4	15.6	< 0.0001
Duration of dialysis, years	4 [2–8]	5 [2–10]	6 [3–12]	7 [4–13]	< 0.0001
Presence of DM, %	37.1	37.5	36.3	34.4	< 0.0001
**Comorbid CVD, %**	31.1	28.9	26.1	21.8	< 0.0001
Coronary artery disease	8.9	8.9	8.2	7.2	
Ischemic stroke	19.7	17.3	15.0	11.5	
Hemorrhagic stroke	5.9	5.8	5.1	4.4	
Limb amputation	3.2	3.4	3.0	3.0	
Body mass index	20.0 ± 3.2	20.8 ± 3.3	21.4 ± 3.4	22.8 ± 3.9	< 0.0001
Dialyzer surface area, m^2^	1.17 ± 0.17	1.50 ± 0	1.75 ± 0.10	2.15 ± 0.13	< 0.0001
**Type of dialyzer**					< 0.0001
Low-flux	3.3	0.6	0.1	0.1	
Medium-flux	2.3	1.3	0.3	0.2	
High-flux	6.4	5.3	2.1	1.7	
Super high-flux	88.0	92.8	97.5	98.0	
Dialysis time, min	224 ± 34	232 ± 30	238 ± 28	246 ± 30	< 0.0001
Ultrafiltration rate, mL/h/kg	11.2 ± 5.4	11.4 ± 4.9	11.4 ± 4.5	11.5 ± 4.2	< 0.0001
β_2_-microglobulin, mg/L	26.1 ± 8.0	26.4 ± 7.2	26.9 ± 6.7	27.4 ± 6.3	< 0.0001
Kt/V	1.40 ± 0.30	1.44 ± 0.29	1.44 ± 0.28	1.43 ± 0.27	< 0.0001
Hemoglobin, g/dL	10.3 ± 1.3	10.5 ± 1.3	10.5 ± 1.2	10.7 ± 1.2	< 0.0001
Serum albumin, g/dL	3.5 ± 0.5	3.6 ± 0.4	3.7 ± 0.4	3.8 ± 0.4	< 0.0001
Calcium, mg/dL	8.8 ± 0.9	8.9 ± 0.8	8.9 ± 0.8	9.0 ± 0.8	< 0.0001
Phosphate, mg/dL	4.9 ± 1.4	5.1 ± 1.4	5.3 ± 1.4	5.6 ± 1.5	< 0.0001
Intact-PTH (pg/mL)	106 [52–187]	112 [56–192]	115 [58–194]	128 [68–212]	< 0.0001
C-reactive protein, mg/dL	0.1 [0.1–0.5]	0.1 [0.1–0.4]	0.1 [0.1–0.3]	0.1 [0.1–0.3]	< 0.0001
nPCR, g/kg/day	0.82 ± 0.19	0.86 ± 0.18	0.88 ± 0.17	0.90 ± 0.17	< 0.0001
SCI, mg/kg/day	73.0 ± 20.6	85.9 ± 21.3	96.7 ± 21.5	109 ± 22.1	< 0.0001
**Outcome variable**					
Death, %	35.3	25.1	20.0	12.3	< 0.0001

Data are expressed as mean ± standard deviation (SD), number (%), or median [interquartile range]. S group, small dialyzer surface area, < 1.5 m^2^; M group, medium dialyzer surface area, 1.5 m^2^; L group, large dialyzer surface area, 1.6 to < 2.0 m^2^; XL group, extra-large dialyzer surface area, ≥ 2.0 m^2^.

*CVD* cardiovascular disease, *DM* diabetes mellitus, *nPCR* normalized protein catabolic rate, *PTH* parathyroid hormone, *SCI* simplified creatinine index.

### Associations between dialyzer surface area and all-cause mortality

**Figure 2:**
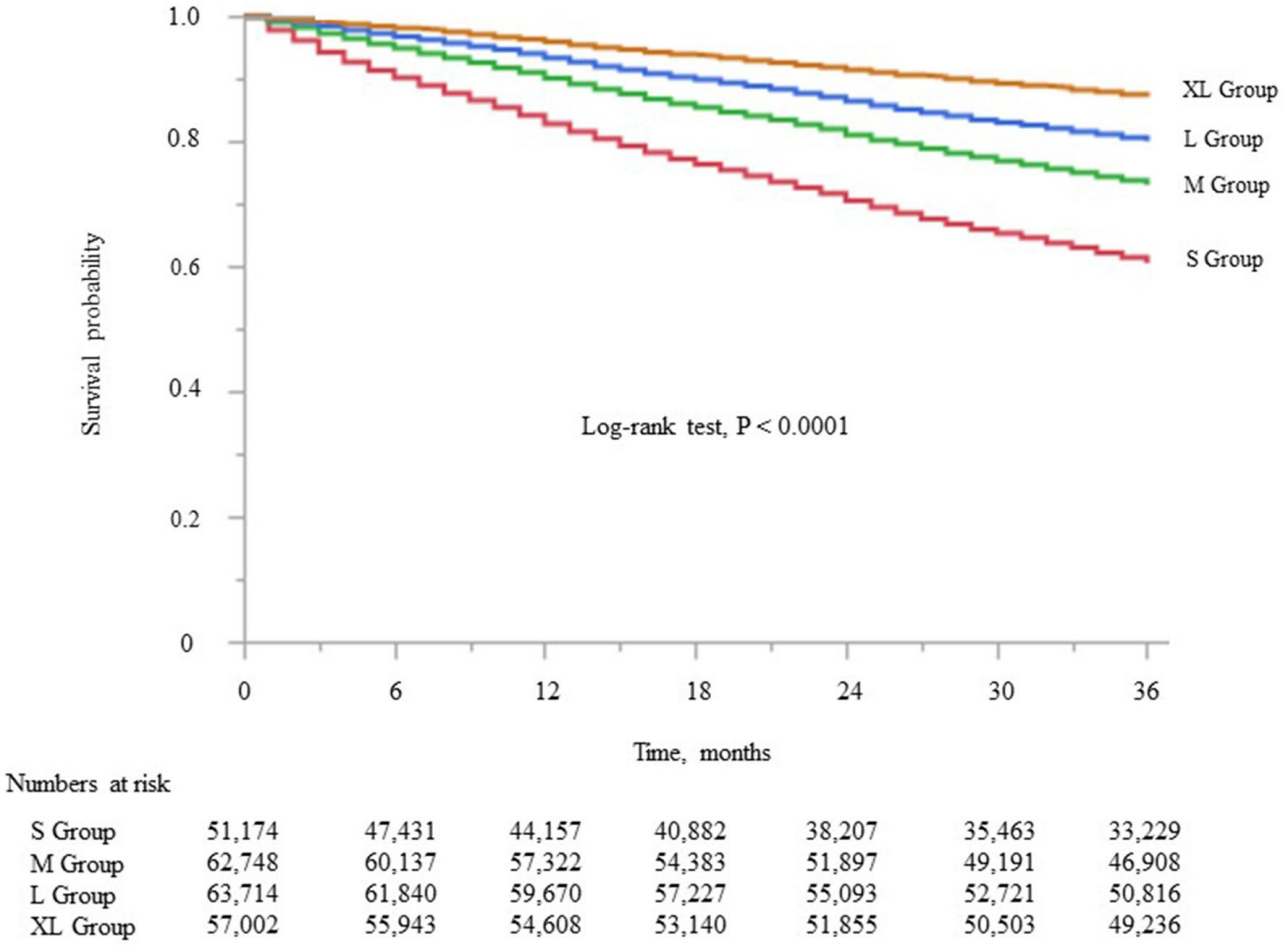
Kaplan–Meier survival curve for all-cause mortality stratified according to dialyzer surface area. S group, small dialyzer surface area, < 1.5 m^2^; M group, medium dialyzer surface area, 1.5 m^2^; L group, large dialyzer surface area, 1.6 to < 2.0 m^2^; XL group, extra-large dialyzer surface area, ≥ 2.0 m^2^.

Kaplan–Meier analysis showed steady deterioration in survival with decreasing dialyzer surface area (log-rank test, *P* < 0.0001; Fig. 2). Compared with the M (reference) group, the S group had a higher unadjusted HR for all-cause mortality (1.61, CI 1.57–1.65). In contrast, the L group and XL group showed lower unadjusted HRs for all-cause mortality (0.71, CI 0.69–0.72 and 0.44, CI 0.43–0.45, respectively; Supplementary Table 4).

**Figure 3:**
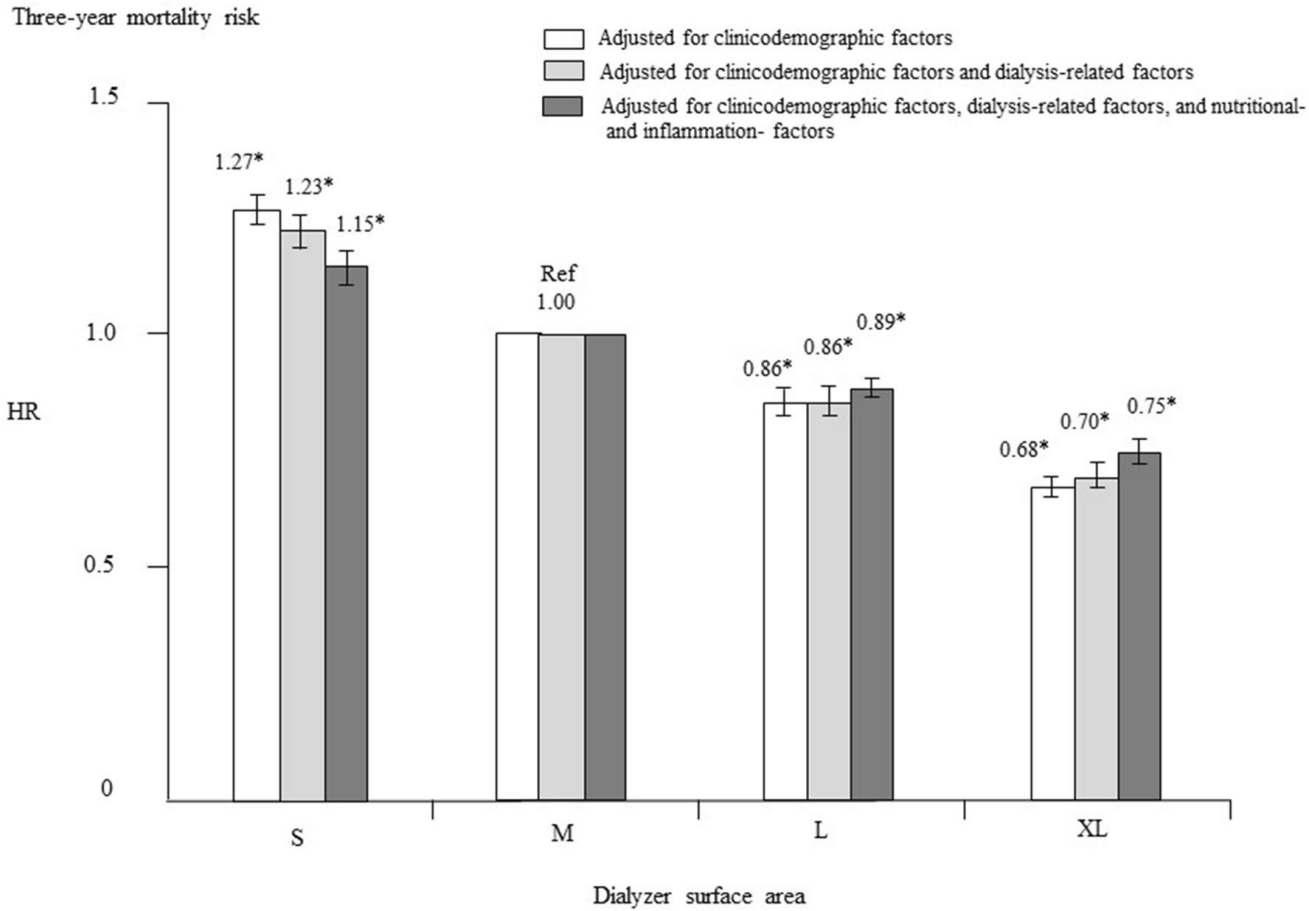
Hazard ratios (HRs) for all-cause mortality in 234,638 patients undergoing hemodialysis according to dialyzer surface area, determined using standard Cox proportional hazards regression analysis. White bars are adjusted for basic factors, including age, sex, dialysis vintage, presence/absence of diabetes mellitus, and presence/absence of cardiovascular complications. Gray bars are adjusted for dialysis-related factors, as assessed by the Kt/V value, β_2_-microglobulin level, ultrafiltration rate, dialysis time, and type of dialyzer in addition to basic factors. Dark gray bars are adjusted for basic factors, dialysis-related factors, and nutrition-related and inflammation-related factors, including body mass index, C-reactive protein, hemoglobin, calcium, phosphate, intact parathyroid hormone, and serum albumin levels, normalized protein catabolic rate, and simplified creatinine index. *P < 0.0001, versus M (reference) group. Error bars correspond to the 95% confidence intervals. S group, small dialyzer surface area, < 1.5 m^2^; M group, medium dialyzer surface area, 1.5 m^2^; L group, large dialyzer surface area, 1.6 to < 2.0 m^2^; XL group, extra-large dialyzer surface area, ≥ 2.0 m^2^.

Figure 3 shows the adjusted HR for all-cause mortality in each group. After adjustment for basic factors, including age, sex, duration of dialysis, history of CVD, and presence or absence of DM, the HRs in the L group and XL group, compared with the M (reference) group, were 0.86 (CI 0.84–0.88) and 0.68 (CI 0.66–0.70), respectively. After adjustment for basic and dialysis-related factors, including Kt/V, β2MG, ultrafiltration rate, hemodialysis time, and dialyzer type, the HRs in the L group and XL group, compared with the M group, were 0.86 (CI 0.84–0.89) and 0.70 (CI 0.68–0.73), respectively. Finally, after adjustment for basic, dialysis-related, nutrition-related, and inflammation-related factors (including BMI, hemoglobin, nPCR, SCI, and serum albumin and CRP levels), the L group and XL group had significantly lower HRs (0.89, CI 0.87–0.92, *P* < 0.0001 and 0.75, CI 0.72–0.78, *P* < 0.0001, respectively). However, the hazard ratio (HR) was consistently and significantly higher in the S group than in the M group (after adjustment for all confounders, HR 1.15, CI 1.12–1.19, *P* < 0.0001).

When the causes of death were categorized as CV-related or non-CV-related, Kaplan–Meier analysis showed that survival deteriorated steadily as the dialyzer surface area decreased in both groups (log-rank test, both *P* < 0.0001; Supplementary Figs. 1 and 2). Compared with the M (reference) group, the S group had higher adjusted HRs (95% CI) for both CV mortality and non-CV mortality. In contrast, the L group and XL group showed lower adjusted HRs for both CV and non-CV mortality (Supplementary Tables 5 and 6).

**Figure 4:**
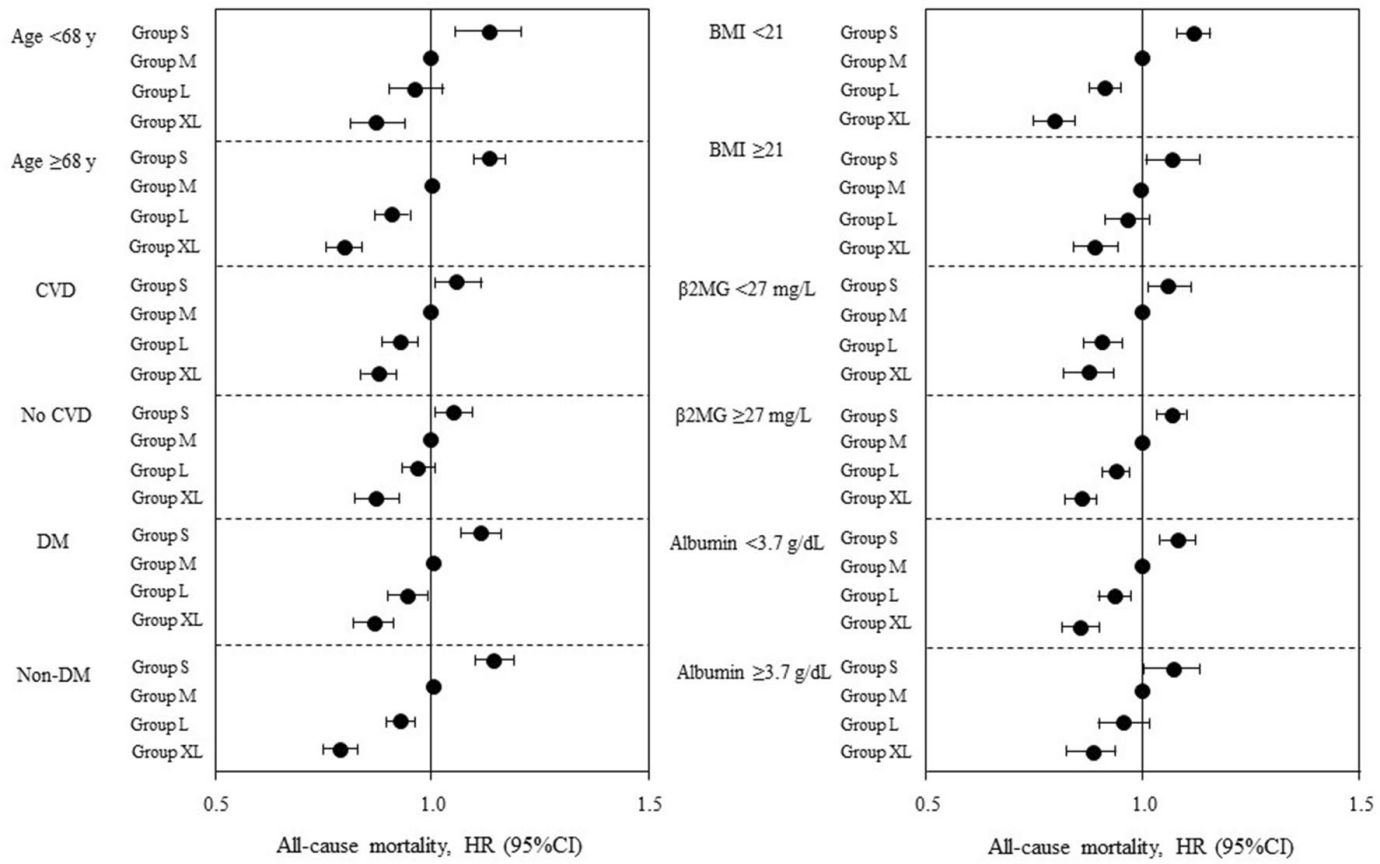
Sensitivity analyses of the association between dialyzer surface area and all-cause mortality stratified by median values for age, body mass index, β2-microglobulin, serum albumin, and comorbid cardiovascular disease and diabetes mellitus at baseline. Circles indicate the adjusted hazard ratio (HR) for mortality and the error bars indicate the 95% confidence interval (CI). The HR for mortality (95% CI) was derived from Cox proportional hazards models adjusted for all covariate values, including basic factors, dialysis-related factors, and nutrition- and inflammation-related factors. S group, small dialyzer surface area, < 1.5 m^2^; M group, medium dialyzer surface area, 1.5 m^2^; L group, large dialyzer surface area, 1.6 to < 2.0 m^2^; XL group, extra-large dialyzer surface area, ≥ 2.0 m^2^. *β2MG*, β_2_-microglobulin; *BMI* body mass index, *DM* diabetes mellitus.

**Figure 5:**
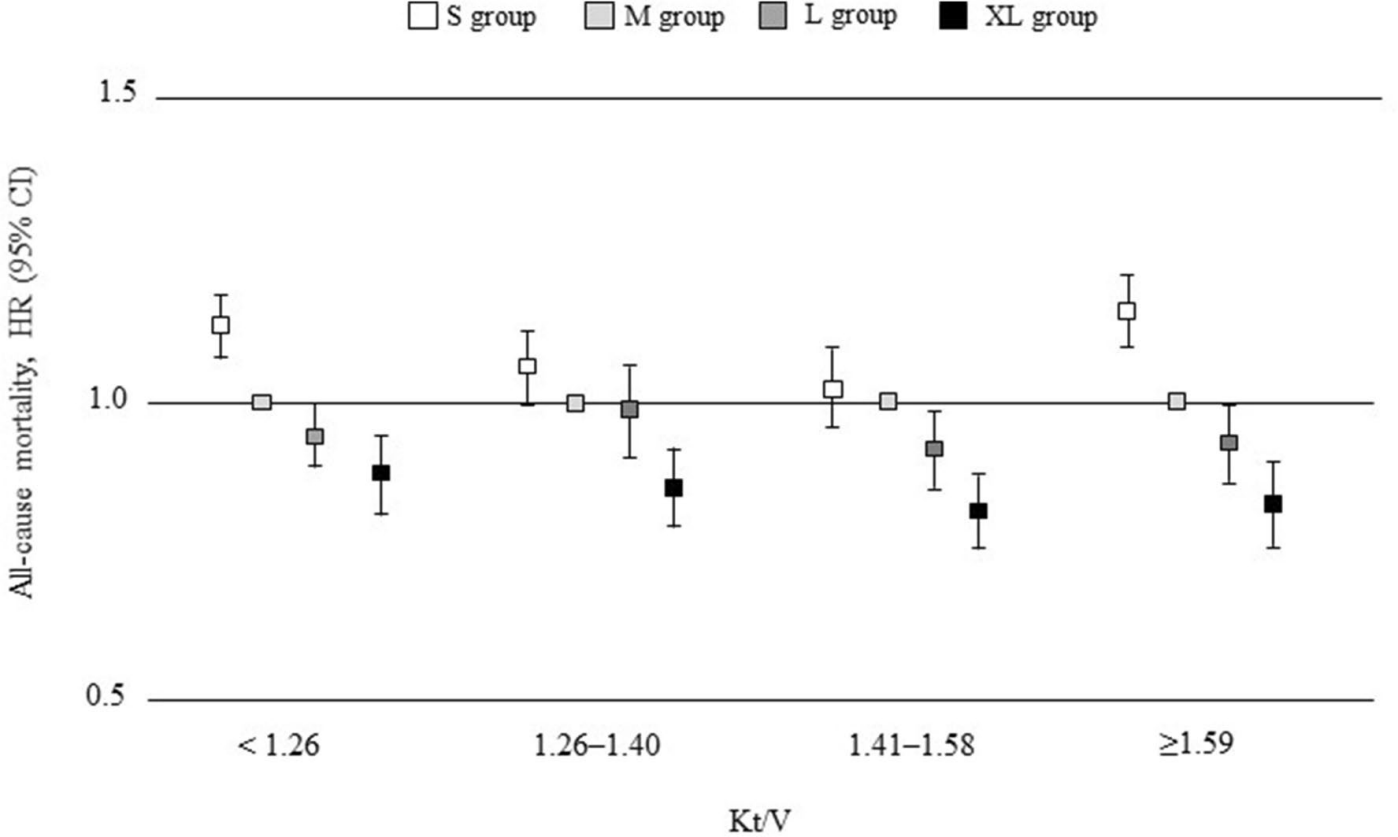
Adjusted hazard ratios for all-cause mortality associated with dialyzer surface area stratified by Kt/V quartile. Squares indicate the adjusted hazard ratios (HRs) for mortality and the error bars indicate the 95% confidence intervals (CIs). The HR for mortality (95% CI) was derived from Cox proportional hazards models adjusted for all covariate values, including basic factors, dialysis-related factors, and nutrition- and inflammation-related factors. S group, small dialyzer surface area, < 1.5 m^2^; M group, medium dialyzer surface area, 1.5 m^2^; L group, large dialyzer surface area, 1.6 to < 2.0 m^2^; XL group, extra-large dialyzer surface area, ≥ 2.0 m^2^.

The sensitivity analysis yielded identical results. After adjustment by all covariates, the risk of all-cause death was higher in the S group regardless of age, CVD, BMI, β2MG, or serum albumin (Fig. 4 and Supplementary Table 7). Analysis to examine the relationship between dialyzer surface area and all-cause mortality after adjustment for covariates in the four dialyzer-flux categories revealed no significant difference in mortality regardless of dialyzer surface area in the low-flux dialyzer group. However, the S group had significantly higher adjusted HRs and the XL group had significantly lower adjusted HRs in the medium, high, and super high-flux dialyzer groups (Table 3). Analysis in the Cox proportional hazards model revealed that both dialyzer surface area and Kt/V were significantly and independently associated with all-cause mortality after adjusting for covariates. Adjusted associations between Kt/V and mortality varied across the dialyzer surface area groups (P_interaction_ = 0.001). A smaller dialyzer surface area had no significant impact on the association between a Kt/V of 1.26–1.58 and mortality. In contrast, the XL group had a significantly lower adjusted mortality risk regardless of Kt/V (Fig. 5 and Supplementary Table 8).

**Table 3:** Hazard ratios (with 95% confidence intervals) for all-cause mortality according to dialyzer surface area and all-cause mortality classified by dialyzer flux type.

Group	n	Unadjusted				Adjusted for basic factors^a^						Adjusted for basic factors and dialysis-related factors^b^						Adjusted for basic factors, dialysis-related factors, and nutrition/inflammation-related factors^c^		
		HR	95% CI	*P* value		HR		95% CI		*P* value		HR		95% CI		*P* value		HR	95% CI	*P* value
**Low-flux dialyzer**																				
S	1,690	1.19	1.01–1.40	0.035		1.18		1.00–1.40		0.049		1.16		0.98–1.40		0.086		1.05	0.88–1.26	0.559
M	396	1.00	Reference	–		1.00		Reference		–		1.00		Reference		–		1.00	Reference	–
L	64	0.80	0.51–1.22	0.328		0.93		0.59–1.42		0.769		0.95		0.59–1.47		0.846		1.09	0.67–1.67	0.709
XL	50	0.49	0.26–0.83	0.007		0.66		0.35–1.13		0.138		0.69		0.35–1.22		0.222		0.83	0.39–1.54	0.637
**Medium-flux dialyzer**																				
S	1,182	1.39	1.22–1.60	< 0.0001		1.34		1.17–1.55		< 0.0001		1.41		1.20–1.64		< 0.0001		1.49	1.22–1.83	< 0.0001
M	800	1.00	Reference	–		1.00		Reference		–		1.00		Reference		–		1.00	Reference	–
L	162	0.91	0.68–1.19	0.523		1.05		0.78–1.38		0.734		1.00		0.73–1.36		0.981		1.46	0.96–2.15	0.073
XL	129	0.42	0.27–0.62	< 0.0001		0.57		0.37–0.85		0.005		0.65		0.41–0.97		0.035		0.46	0.19–0.93	0.003
**High-flux dialyzer**																				
S	3,295	1.53	1.40–1.67	< 0.0001		1.32		1.21–1.44		< 0.0001		1.31		1.20–1.44		< 0.0001		1.26	1.12–1.42	0.0001
M	3,292	1.00	Reference	–		1.00		Reference		–		1.00		Reference		–		1.00	Reference	–
L	1,341	0.84	0.74–0.95	0.007		0.90		0.79–1.03		0.132		0.94		0.82–1.07		0.981		0.96	0.81–1.14	0.681
XL	978	0.46	0.38–0.55	< 0.0001		0.64		0.53–0.76		< 0.0001		0.67		0.55–0.80		< 0.0001		0.74	0.57–0.94	0.018
**Super high-flux dialyzer**																				
S	45,007	1.48	1.45–1.51	< 0.0001		1.23		1.20–1.26		< 0.0001		1.23		1.20–1.26		< 0.0001		1.07	1.03–1.10	< 0.0001
M	58,260	1.00	Reference	–		1.00		Reference		–		1.00		Reference		–		1.00	Reference	–
L	62,147	0.78	0.76–0.80	< 0.0001		0.88		0.86–0.90		< 0.0001		0.88		0.85–0.90		< 0.0001		0.93	0.90–0.96	< 0.0001
XL	55,845	0.49	0.47–0.50	< 0.0001		0.71		0.69–0.74		< 0.0001		0.71		0.69–0.30		< 0.0001		0.84	0.81–0.88	< 0.0001

S group, small dialyzer surface area, < 1.5 m^2^; M group, medium dialyzer surface area, 1.5 m^2^; L group, large dialyzer surface area, 1.6 to < 2.0 m^2^; XL group, extra-large dialyzer surface area, ≥ 2.0 m^2^.

^a^Adjusted for age, sex, duration of dialysis, presence or absence of diabetes, and cardiovascular disease. ^b^Adjusted for basic factors and dialysis-related factors, including Kt/V, β_2_-microglobulin level, ultrafiltration rate, and dialysis time. ^c^Adjusted for basic factors and dialysis-related factors, C-reactive protein, hemoglobin, normalized protein catabolic rate, serum albumin, body mass index, and simplified creatinine index.

*CI* confidence interval, *HR* hazard ratio.

### Propensity score matching analysis

**Figure 6:**
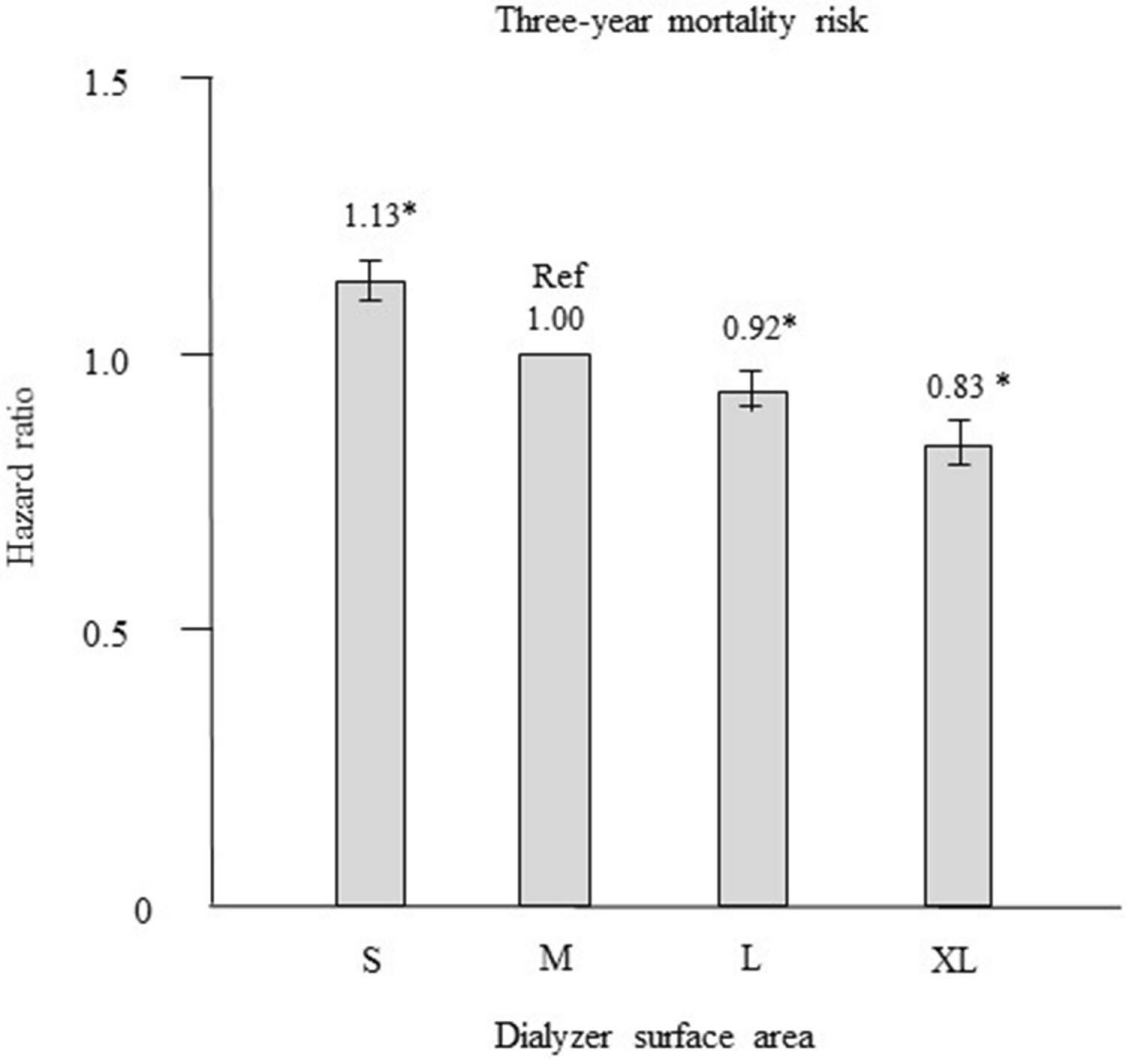
Hazard ratios for all-cause mortality for the three dialyzer surface area groups versus the reference group after propensity score matching using a Cox proportional hazards regression model. *P < 0.0001 versus M group. Error bars correspond to 95% confidence intervals. S group, small dialyzer surface area, < 1.5 m^2^; M group, medium dialyzer surface area, 1.5 m^2^; L group, large dialyzer surface area, 1.6 to < 2.0 m^2^; XL group, extra-large dialyzer surface area, ≥ 2.0 m^2^.

Patients in the M group were matched with those in the other groups in a 1:1 ratio according to propensity scores. After propensity score matching, 7336, 8639, and 5242 patient pairs were matched in the S, L, and XL groups, respectively. Table 4 shows the patient characteristics and clinical data at baseline in the M group and in each corresponding group after propensity score matching. No significant differences were noted for any of the variables. Compared with the M group, the S group had a significantly higher HR for all-cause mortality (1.13, CI 1.10–1.16, *P* < 0.0001), whereas the L and XL groups had significantly lower HRs (0.92, CI 0.89–0.95 and 0.83, 0.79–0.87, both *P* < 0.0001; Fig. 6).

**Table 4:** Baseline characteristics after propensity score matching between the M (reference) group and other groups.

Variable	Matched			Matched			Matched		
	S group	M group	*P* value	L group	M group	*P* value	XL group	M group	*P* value
n (%)	7336	7336	–	8639	8639	–	5242	5242	–
Age (years)	69.7 ± 11.4	69.7 ± 10.4	0.880	66.2 ± 10.9	66.1 ± 11.4	0.806	63.7 ± 10.4	63.8 ± 11.6	0.389
Female sex (%)	54.4	53.9	0.567	35.0	35.6	0.723	29.5	29.8	0.732
Duration of dialysis (years)	4 [2–9]	4 [2–9]	0.708	6 [3–11]	6 [3–11]	0.529	6 [3–12]	6 [3–11]	0.365
Presence of DM (%)	37.3	38.3	0.402	36.6	37.1	0.982	37.0	36.7	0.881
**History of CVD (%)**	29.5	30.0	0.559	26.0	26.1	0.822	25.9	25.6	0.754
Coronary artery disease	8.4	8.5	0.720	7.6	7.8	0.530	7.7	7.9	0.662
Ischemic stroke	18.6	18.4	0.824	15.7	15.5	0.801	15.2	15.2	0.935
Hemorrhagic stroke	5.1	5.5	0.360	5.0	5.0	0.834	4.9	4.5	0.407
Limb amputation	3.2	3.4	0.654	2.5	2.5	0.922	3.1	3.0	0.735
Body mass index, kg/m^2^	20.5 ± 3.3	20.6 ± 3.3	0.226	21.1 ± 3.3	21.1 ± 3.3	0.981	21.6 ± 3.4	21.6 ± 3.5	0.855
β2MG (mg/L)	26.3 ± 7.9	26.3 ± 7.2	0.993	27.5 ± 7.2	27.5 ± 7.4	0.511	27.9 ± 6.7	27.9 ± 6.8	0.678
Kt/V	1.42 ± 0.34	1.42 ± 0.34	0.221	1.45 ± 0.31	1.45 ± 0.32	0.532	1.44 ± 0.31	1.45 ± 0.33	0.252
**Type of dialyzer (%)**			0.836			0.169			0.882
Low-flux	1.1	1.0		0.3	0.4		0.2	0.3	
Medium-flux	1.7	1.6		0.3	0.4		0.5	0.4	
High-flux	6.0	6.1		3.5	4.0		3.0	3.0	
Super high-flux	91.2	91.3		95.9	95.2		96.3	96.3	
Hemoglobin (g/dL)	10.5 ± 1.2	10.5 ± 1.2	0.804	10.6 ± 1.2	10.6 ± 1.2	0.954	10.6 ± 1.2	10.5 ± 1.2	0.504
Serum albumin (g/dL)	3.6 ± 0.4	3.6 ± 0.4	0.252	3.7 ± 0.4	3.7 ± 0.4	0.509	3.7 ± 0.4	3.7 ± 0.4	0.663
Calcium (mg/dL)	8.8 ± 0.8	8.8 ± 0.8	0.776	8.9 ± 0.8	8.9 ± 0.8	0.594	8.9 ± 0.8	8.9 ± 0.8	0.507
Phosphate (mg/dL)	5.0 ± 1.4	5.0 ± 1.4	0.632	5.2 ± 1.4	5.2 ± 1.4	0.369	5.4 ± 1.5	5.4 ± 1.5	0.817
Intact-PTH (pg/mL)	111 [56–187]	110 [55–191]	0.226	114 [57–198]	112 [56–188]	0.682	122 [61–206]	124 [66–207]	0.987
CRP (mg/dL)	0.1 [0.1–0.4]	0.1 [0.1–0.4]	0.897	0.1 [0.1–0.3]	0.1 [0.1–0.3]	0.931	0.1 [0.1–0.3]	0.1 [0.1–0.3]	0.950
nPCR (g/kg/day)	0.84 ± 0.18	0.84 ± 0.17	0.947	0.86 ± 0.17	0.86 ± 0.17	0.787	0.87 ± 0.17	0.87 ± 0.17	0.728
SCI (mg/kg/day)	81.8 ± 20.0	81.9 ± 19.5	0.733	91.8 ± 20.0	91.8 ± 20.3	0.919	97.1 ± 19.5	96.9 ± 20.2	0.070

Data are expressed as mean ± standard deviation (SD), number (%), or median [interquartile range]. S group, small dialyzer surface area, < 1.5 m^2^; M group, medium dialyzer surface area, 1.5 m^2^; L group, large dialyzer surface area, 1.6 to < 2.0 m^2^; XL group, extra-large dialyzer surface area, ≥ 2.0 m^2^. β2MG, β_2_-microglobulin.

*CRP* C-reactive protein, *CVD* cardiovascular disease, *DM* diabetes mellitus, *nPCR* normalized protein catabolic rate, *PTH* parathyroid hormone, *SCI* simplified creatinine index.

## Discussion

In this study, analysis of data from a large registry of 234,638 Japanese patients on hemodialysis over a 3-year period revealed a dose–response association between the dialyzer surface area and all-cause mortality. When mortality was compared between four groups according to dialyzer surface area, after adjustment for predictive factors and propensity score matching, the HR for all-cause mortality was significantly lower in the group with a larger dialyzer surface area, even in the same Kt/V category. A major strength of this study is the large sample size and inclusion of all types of dialyzers currently available in Japan. This study is the first to indicate the potential improvement in mortality risk by using dialyzers with larger surface area.

Previously, the large randomized controlled HEMO study found no significant difference in mortality between patients receiving low-dose dialysis and those on high-dose dialysis, with respective Kt/V values of 1.32 and 1.71^17^. Increases in the dialysis dose and clearance of small-molecular-weight substances were found not to be associated with improved outcomes in the hemodialysis patients; however, the dialysis prescription was targeted to have high efficiency during a short dialysis treatment time. In contrast, in the Dialysis Outcomes and Practice Patterns Study, a longer dialysis treatment time and higher Kt/V were independently associated with lower mortality^8^. A longer treatment time was associated with lower mortality at any Kt/V level. Furthermore, at higher Kt/V levels, even more benefit was seen with a longer treatment time than with the same treatment time for a lower Kt/V level. Therefore, there was a synergistic relationship between Kt/V and treatment time in terms of lower mortality. In Japan, the target Kt/V is achieved by prolonging the treatment time, whereas in the US and Europe it is achieved by increasing the blood flow rate or dialyzer surface area^8^. Therefore, an increased dialyzer surface area might be associated with further reduction of mortality in Japanese patients on dialysis. Although the present study excluded patients whose treatment time was < 2 h, a significant association was found between a larger dialyzer surface area and a lower mortality risk.

The blood flow rate and dialyzer surface area are important factors for dialysis efficiency. The blood flow rate is significantly lower in patients on hemodialysis in Japan compared to those in other countries because more than 90% of Japanese patients have an arteriovenous fistula for vascular access^18^. Use of an arteriovenous fistula was shown to be superior to an arteriovenous graft and central venous catheter. Median blood flow rates were found to vary across regions, at 200 mL/min in Japan, 300 mL/min in Europe, Australia, and New Zealand, and 400 mL/min in North America^19^. However, a significant association has been found between a higher blood flow rate and a lower mortality risk in patients on hemodialysis, even in Japan^3^. Therefore, given the lack of an association between an increased dialysis dose based on the Kt/V and a better prognosis, more comprehensive assessment of treatment time, blood flow rate, and dialyzer surface area is needed to improve the outlook in patients on hemodialysis.

Use of dialyzers with a smaller surface area in Japan may reflect the fact that BMI is significantly lower in Japanese patients on hemodialysis compared with their counterparts in other countries^20^. Although there have been no published comparisons of dialyzer surface area between countries, mean area might be associated with body surface area or BMI. Approximately 90% of patients in the HEMO study were treated with a dialyzer surface area of 1.8–2.1 m^2^^17^. However, in the present study, dialyzers with a larger surface area were found to be superior regardless of BMI. The hemodialysis prescription in Japan is characterized by a lower blood flow rate, smaller dialyzer surface area, lower Kt/V, and longer dialysis treatment time. Furthermore, super high-flux or HPM dialyzers are more likely to be used in Japan^21^. Recently, not only middle-molecular-weight toxins, such as β2MG, but also high-molecular-weight toxins, such as α1-microglobulin (α1-MG) and protein-bound uremic toxins, have been targeted for removal in hemodialysis patients, which might improve prognosis^22,23^. Super high-flux dialyzers have larger pores than high-flux dialyzers, which means that they can remove small, medium, and large molecules, including low-molecular-weight proteins and small amounts of albumin^24,25^. The optimal pore size of super high-flux dialyzers should prevent loss of > 3 g of albumin per session when using the standard hemodialysis procedure in Japan of a blood flow rate of 200 mL/min and a dialysate flow rate of 500 mL/min^3,24^. Our findings suggest that the significance of a larger dialyzer surface area is independent of Kt/V and that a larger surface area could further enhance the beneficial effect of a given Kt/V. Although super high-flux dialyzers might increase the removal of middle-molecular-weight, high-molecular-weight, and protein-bound uremic toxins in relation to the surface area, low-flux dialyzers might not be able to increase such removal even if they have a larger surface area. In addition, internal filtration would increase with the larger surface area of super high-flux dialyzers, which might lead to increased removal of medium- to high- molecular weight solutes^26,27^. Thus, in patients who can tolerate larger dialyzers, use of super high-flux dialyzers with a larger surface area may contribute to lower mortality even when the blood flow rate and Kt/V are low. However, further studies are needed to confirm whether the larger surface area afforded by super high-flux dialyzers is associated with better prognosis due to the removal of larger amounts of middle-sized substances and protein-bound uremic toxins, because we were not able to measure the clearance of these toxins.

Malnutrition and inflammation also predict mortality in patients on dialysis^28–30^. In the present study, after adjustment for basic factors, a smaller dialyzer surface area was associated with higher mortality, whereas a larger surface area was associated with lower mortality. This trend was not changed after further adjustment for dialysis dose. This means that there was no significant association between Kt/V and mortality. When further adjusted for nutrition-related and inflammation-related factors, the mortality rate was lower in the smaller surface area group and greater in the larger surface area group. This finding suggests that the higher mortality risk in the smaller surface area group was associated with poor nutritional status and that the lower mortality risk in the larger surface area group was associated with good nutritional status. Therefore, increasing the dialyzer surface area is recommended for patients with good nutritional status. However, mortality was significantly lower in the larger surface area group when patients were stratified by serum albumin level and BMI. Further studies are needed to determine whether use of dialyzers with a larger surface area might be beneficial even in patients with malnutrition.

This study has several limitations. First, given that we needed to exclude data for 11.3% of potential study participants due to missing information on dialyzer surface area, there may have been some degree of selection bias as a result of variations in the dialyzers used and mortality rates between facilities due to differences in practice and patient populations. Second, patients in the smaller surface area groups had poorer nutritional status, a higher rate of comorbid CVD, and higher rate of using low-flux dialyzers, which could have introduced further selection bias. However, we confirmed the superiority of the larger surface area dialyzers after propensity score matching analysis. Third, unknown or unmeasured confounders may have affected the association between dialyzer surface area and mortality. We could not collect data on comorbidities except for DM and CVD, or Charlson comorbidity index scores such as for heart failure, chronic obstructive pulmonary disease, and malignancy. Those uncontrolled comorbidities may be possible confounders. The present study is observational in nature and we could not analyze cause-effect relationships, so we must be careful in interpreting the results. We also had no data available on residual kidney function. However, in 2007, it was reported that the reduction in kidney function after initiating hemodialysis was 2.0 mL/min/year and the mean estimated glomerular filtration rate at dialysis initiation was 6.5 mL/min/1.73 m^2^ in Japanese dialysis patients^31^. Therefore, the impact of residual kidney function may have been negligible given that the median dialysis duration was 6 years in the present cohort. In addition, we did not obtain any data on blood pressure, which could be a possible confounder. Dialyzers with a smaller surface area might be more beneficial in patients with hypotension due to impaired cardiac function. To improve the prognosis, protein-bound uremic toxins and middle-sized substances, such as β2MG and α1-MG, are now being targeted for removal in patients on hemodialysis^22,23,32^. Removal of middle-sized substances depends on both dialyzer permeability and treatment modality. Therefore, super high-flux dialyzers with a larger surface area may contribute to greater removal of middle-sized substances that cannot be assessed by the Kt/V.

In conclusion, our findings suggest a significant association between dialyzer surface area and mortality in patients on hemodialysis, and more specifically that the larger surface area of the super high-flux dialyzers might be beneficial. Thus far, dialyzer surface area has not been considered in this regard because it has been thought of as a configuration factor for the Kt/V. However, it should be reconsidered because it might contribute more than the Kt/V value to the mortality risk in patients on hemodialysis. Randomized controlled studies are warranted to determine whether the larger surface area of the super high-flux dialyzers improves outcomes in patients on hemodialysis.

## Supplementary Information

Supplementary Figure 1.

Supplementary Figure 2.

Supplementary Information.

Supplementary Table S1.

Supplementary Table S2.

Supplementary Table S3.

Supplementary Table S4.

Supplementary Table S5.

Supplementary Table S6.

Supplementary Table S7.

Supplementary Table S8.
