# Supplementary figures and images for: Dialyzer surface area is a significant predictor of mortality in patients on hemodialysis: a 3-year nationwide cohort study
Source: Sci Rep. 2021 Oct 18;11:20616. doi: 10.1038/s41598-021-99834-4 (PMC8523692; doi:10.1038/s41598-021-99834-4)

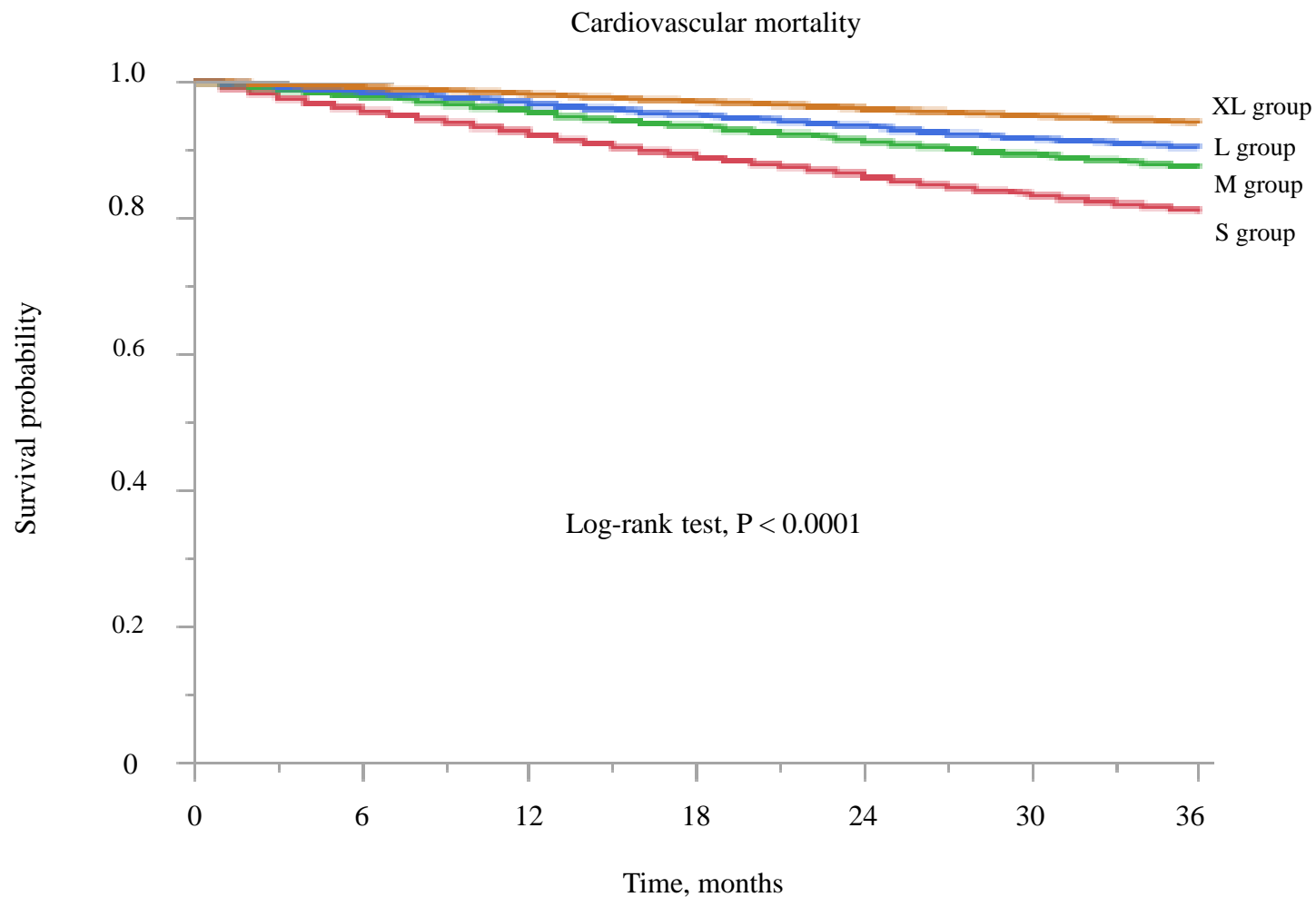

Numbers at risk

|          |        |        |        |        |        |        |        |
|----------|--------|--------|--------|--------|--------|--------|--------|
| S Group  | 40,968 | 39,301 | 37,828 | 36,371 | 35,178 | 33,968 | 32,963 |
| M Group  | 53,806 | 52,626 | 51,359 | 50,036 | 48,890 | 47,680 | 46,675 |
| L Group  | 56,547 | 55,638 | 54,645 | 53,494 | 52,496 | 51,414 | 50,596 |
| XL Group | 52,894 | 52,346 | 51,708 | 50,981 | 50,348 | 49,720 | 49,085 |

Supplement: Supplementary file 1 — Supplementary Figure 1. [file 41598_2021_99834_MOESM1_ESM.pdf]

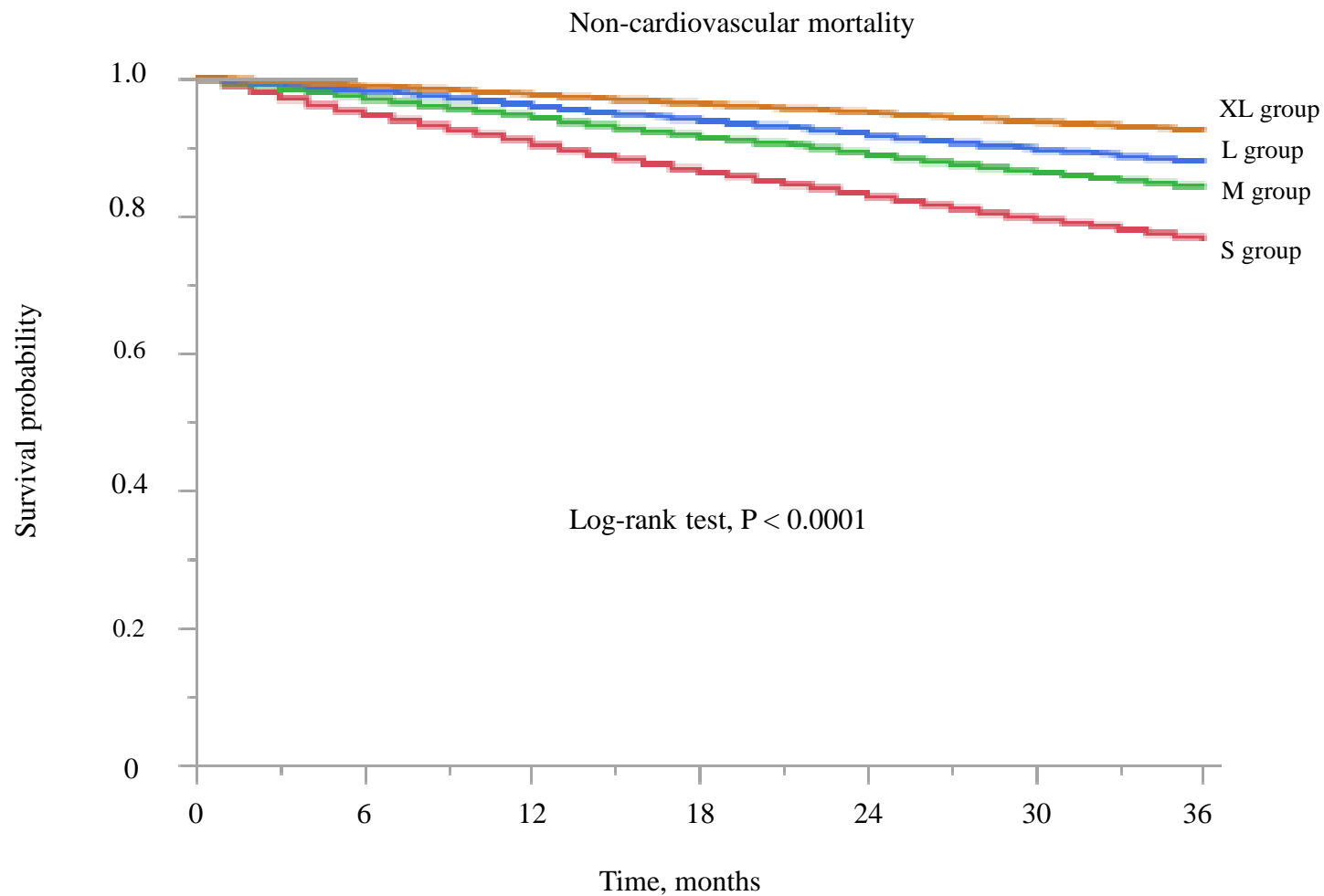

Numbers at risk

|          |        |        |        |        |        |        |        |
|----------|--------|--------|--------|--------|--------|--------|--------|
| S Group  | 43,304 | 41,150 | 39,286 | 37,411 | 35,871 | 34,313 | 33,043 |
| M Group  | 55,921 | 54,396 | 52,742 | 51,034 | 49,622 | 48,064 | 46,733 |
| L Group  | 58,167 | 57,111 | 55,815 | 54,426 | 52,496 | 51,830 | 50,662 |
| XL Group | 53,750 | 53,119 | 52,274 | 51,417 | 50,650 | 49,843 | 49,127 |

Supplement: Supplementary file 2 — Supplementary Figure 2. [file 41598_2021_99834_MOESM2_ESM.pdf]
