# Supplementary material for: Dialyzer surface area is a significant predictor of mortality in patients on hemodialysis: a 3-year nationwide cohort study
Source: Sci Rep. 2021 Oct 18;11:20616. doi: 10.1038/s41598-021-99834-4 (PMC8523692; doi:10.1038/s41598-021-99834-4)
Supplement: Supplementary file 3 — Supplementary Information. [file 41598_2021_99834_MOESM3_ESM.pdf]

Supplementary Figure legends

Supplementary Figure 1. Kaplan–Meier survival curve for cardiovascular mortality stratified by dialyzer surface area. S group, small dialyzer surface area, <1.5 m^2; M group, medium dialyzer surface area, 1.5 m^2; L group, large dialyzer surface area, 1.6 to <2.0 m^2; XL group, extra-large dialyzer surface area, ≥2.0 m^2.

Supplementary Figure 2. Kaplan–Meier survival curve for non-cardiovascular mortality stratified by dialyzer surface area. S group, small dialyzer surface area, <1.5 m^2; M group, medium dialyzer surface area, 1.5 m^2; L group, large dialyzer surface area, 1.6 to <2.0 m^2; XL group, extra-large dialyzer surface area, ≥2.0 m^2.
