# Supplementary material for: Dialyzer surface area is a significant predictor of mortality in patients on hemodialysis: a 3-year nationwide cohort study
Source: Sci Rep. 2021 Oct 18;11:20616. doi: 10.1038/s41598-021-99834-4 (PMC8523692; doi:10.1038/s41598-021-99834-4)
Supplement: Supplementary file 4 — Supplementary Table S1. [file 41598_2021_99834_MOESM4_ESM.docx]

# Supplementary Table 1. Equations for Kt/V, normalized protein catabolic rate, and simplified creatinine

**Calculation of Kt/V**

Kt/V was calculated using Shinzato’s formula [15]:


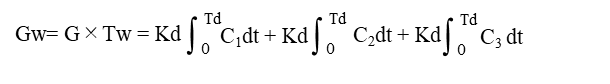


Here, Gw is the urea volume generated over a 1-week period, G is the urea generation rate, Tw is 1 week, Kd is the urea clearance of the dialyzer, K is the dialyzer urea clearance, T is the dialysis time, and C1, C2, and C3 are urea concentrations during the respective dialysis session.

Td, dialysis time;.

ln (CE)－

Ln (CE _L_)×[Kt/V] _H_－ln (CE _H_)×[Kt/V] _L_

[Kt/V]_H_－[Kt/V]_L_

ln (CE _H_)－ln (CE _L_)

[Kt/V] _H_－[Kt/V] _L_

Kt/V =

Here, CE is the post-dialysis serum urea nitrogen given by the formula CE = CS Exp (-Kt/V) + G/K [1-Exp (-Kt/V)], where CS is the pre-dialysis serum urea nitrogen concentration. Moreover, [Kt/V] _L_ = ln (CS/CE), [Kt/V] _H_ = ln [ (CS-Y)/(CE-Y)], Y = (CS－CE) TD / ln (CS/CE) TI_d_, and TI_d_ indicates time between dialysis sessions on weekdays.

**Calculation of normalized protein catabolic rate (nPCR)**

nPCR was calculated using Shinzato’s formula [15]:

G・TI = V・CS－V・CE’

g・TI = (v + Δv) CS－v・CE’

Here, CS and CE’ are serum urea nitrogen concentration before any given dialysis session and after the previous dialysis session, respectively; g and G are urea generation rate calculated with a variable and constant volume of urea distribution, respectively; v is the volume of urea distribution after dialysis session when the change in the volume taken into account, and V is the volume when the change in the volume of urea distribution is ignored; and Δv is the increase in the volume of urea distribution between dialysis sessions.

g/v = (CS’－CE + Δv/v CS’)/TI

PCR = 9.35g + 0.29 v

PCR/IBW = 9.35 × γ × g/v + 0.29 γ

Here, γ is the ratio of the volume of urea distribution relative to the ideal body weight. IBW, ideal body weight.

**Calculation of SCI**

SCI was calculated using the Canaud formula [16]:

SCI (mg/kg/day) = 16.21 + 1.12 × [1 if male; 0 if female] － 0.06 × age (years) － 0.08 ×

spKt/V urea + 0.009 × pre-hemodialysis SCr (μmol/L)
