# Supplementary material for: Dialyzer surface area is a significant predictor of mortality in patients on hemodialysis: a 3-year nationwide cohort study
Source: Sci Rep. 2021 Oct 18;11:20616. doi: 10.1038/s41598-021-99834-4 (PMC8523692; doi:10.1038/s41598-021-99834-4)
Supplement: Supplementary file 5 — Supplementary Table S2. [file 41598_2021_99834_MOESM5_ESM.docx]

# Supplementary Table 2. Definitions and classifications of dialyzer flux type

| International classification | Japanese classification | β2MG clearance (mL/min) | UN clearance (mL/min) | UFR (mL/h/mmHg) |
| --- | --- | --- | --- | --- |
| Low-flux | I | < 10 | ≥ 125 | < 15 |
| Medium-flux | II | ≥ 10-30 | ≥ 150 | ≥ 15 |
| High-flux | III | ≥ 30-50 | ≥ 150 | ≥ 15 |
| Super high-flux | IV | ≥ 50-70 | ≥ 150 | ≥ 50 |
| Super high-flux | V | ≥ 70 | ≥ 150 | ≥ 50 |

β2MG, β_2_-microglobulin; UFR, ultrafiltration rate; UN, urea nitrogen.
