# Supplementary material for: Dialyzer surface area is a significant predictor of mortality in patients on hemodialysis: a 3-year nationwide cohort study
Source: Sci Rep. 2021 Oct 18;11:20616. doi: 10.1038/s41598-021-99834-4 (PMC8523692; doi:10.1038/s41598-021-99834-4)
Supplement: Supplementary file 6 — Supplementary Table S3. [file 41598_2021_99834_MOESM6_ESM.docx]

**Supplementary Table 3**. Hazard ratios and 95% confidence intervals for variables evaluated as potential predictors of mortality among 234,638 patients

| Variable | HR | 95% CI | P-value |
| --- | --- | --- | --- |
| Sex |  |  |  |
| Male | 1.00 | Reference | - |
| Female | 0.94 | 0.923–0.954 | < 0.0001 |
| Age (years) |  |  |  |
| 1-year increase | 1.064 | 1.063–1.065 | < 0.0001 |
| Duration of hemodialysis (years) |  |  |  |
| <2 | 0.973 | 0.945–1.002 | 0.067 |
| ≥2–5 | 1.000 | Reference | - |
| ≥5–10 | 1.060 | 1.038–1.083 | < 0.0001 |
| ≥10–20 | 0.912 | 0.891–0.934 | < 0.0001 |
| ≥20 | 0.814 | 0.786–0.844 | < 0.0001 |
| Primary kidney disease |  |  |  |
| Glomerulonephritis | 1.000 | Reference | - |
| Diabetic nephropathy | 1.486 | 1.458–1.516 | < 0.0001 |
| Nephrosclerosis | 1.549 | 1.503–1.596 | < 0.0001 |
| PKD | 0.855 | 0.808–0.903 | < 0.0001 |
| Other | 1.118 | 1.071–1.168 | < 0.0001 |
| Comorbid CVD |  |  |  |
| No | 1.000 | Reference | - |
| Yes | 2.072 | 2.023–2.110 | < 0.0001 |
| Kt/V |  |  |  |
| <1.0 | 1.447 | 1.404–1.491 | < 0.0001 |
| ≥1.0–1.2 | 1.125 | 1.096–1.155 | < 0.0001 |
| ≥1.2–1.4 | 1.000 | Reference | - |
| ≥1.4–1.6 | 0.942 | 0.919–0.965 | < 0.0001 |
| ≥1.6–1.8 | 0.888 | 0.863–0.915 | < 0.0001 |
| ≥1.8 | 0.762 | 0.735–0.789 | < 0.0001 |
| Dialysis time |  |  |  |
| 1-hr increase | 0.565 | 0.556-0.574 | < 0.0001 |
| Ultrafiltration rate |  |  |  |
| 1-mL/h/kg increase | 1.004 | 1.002-1.005 | < 0.0001 |
| β_2_-microglobulin (mg/L) |  |  |  |
| 1-mg/L increase | 1.022 | 1.021–1024 | < 0.0001 |
| C-reactive protein (mg/dL) |  |  |  |
| 1-mg/dL increase | 1.102 | 1.100–1.104 | < 0.0001 |
| Hemoglobin (g/dL) |  |  |  |
| 1-g/dL increase | 0.813 | 0.808–0.819 | < 0.0001 |
| Body mass index |  |  |  |
| 1-unit increase | 0.891 | 0.889–0.894 | < 0.0001 |
| Serum albumin (g/dL) |  |  |  |
| <3.0 | 4.996 | 4.872–5.122 | < 0.0001 |
| ≥3.0–3.5 | 2.158 | 2.115–2.202 | < 0.0001 |
| ≥3.5–4.0 | 1.000 | Reference | - |
| ≥4.0–4.5 | 0.559 | 0.543–0.576 | < 0.0001 |
| ≥4.5 | 0.435 | 0.386–0.490 | < 0.0001 |
| nPCR (g/kg/day) |  |  |  |
| <0.6 | 2.651 | 2.573–2.732 | < 0.0001 |
| ≥0.6–0.8 | 1.431 | 1.402–1.460 | < 0.0001 |
| ≥0.8–1.0 | 1.000 | Reference | - |
| ≥1.0–1.2 | 0.821 | 0.799–0.844 | < 0.0001 |
| ≥1.2 | 0.883 | 0.839–0.929 | < 0.0001 |
| SCI (mg/kg/day) |  |  |  |
| 1 mg/kg/day increase | 0.973 | 0.972–0.974 | < 0.0001 |

CVD, cardiovascular disease; nPCR, normalized protein catabolic rate; PKD, polycystic kidney disease; SCI, simplified creatinine index
