# Supplementary material for: Dialyzer surface area is a significant predictor of mortality in patients on hemodialysis: a 3-year nationwide cohort study
Source: Sci Rep. 2021 Oct 18;11:20616. doi: 10.1038/s41598-021-99834-4 (PMC8523692; doi:10.1038/s41598-021-99834-4)
Supplement: Supplementary file 7 — Supplementary Table S4. [file 41598_2021_99834_MOESM7_ESM.docx]

**Supplementary Table 4.** Hazard ratios (95% confidence intervals) for all-cause mortality according to dialyzer surface area in 234,638 patients on maintenance hemodialysis, determined using a standard Cox proportional hazards regression model

| Group | Unadjusted | | |  | Adjusted for basic factors^a^ | | |  | Adjusted for basic factors and dialysis-related factors^b^ | | |  | Adjusted for basic, dialysis-related, and nutrition/inflammation-related factors^c^ | | |
| --- | --- | --- | --- | --- | --- | --- | --- | --- | --- | --- | --- | --- | --- | --- | --- |
|  | HR | 95% CI | P-value |  | HR | 95% CI | P-value |  | HR | 95% CI | P-value |  | HR | 95% CI | P-value |
| S | 1.61 | 1.57–1.65 | < 0.0001 |  | 1.27 | 1.24–1.30 | < 0.0001 |  | 1.23 | 1.23–1.29 | < 0.0001 |  | 1.15 | 1.12–1.19 | < 0.0001 |
| M | 1.00 | Reference | – |  | 1.00 | Reference | – |  | 1.00 | Reference | – |  | 1.00 | Reference | – |
| L | 0.71 | 0.69–0.72 | < 0.0001 |  | 0.86 | 0.84–0.88 | < 0.0001 |  | 0.86 | 0.84–0.89 | < 0.0001 |  | 0.89 | 0.87–0.92 | < 0.0001 |
| XL | 0.44 | 0.43–0.45 | < 0.0001 |  | 0.68 | 0.66–0.70 | < 0.0001 |  | 0.70 | 0.68–0.73 | < 0.0001 |  | 0.75 | 0.72–0.78 | < 0.0001 |

S group, small dialyzer surface area, <1.5 m^2^; M group, medium dialyzer surface area, 1.5 m^2^; L group, large dialyzer surface area, 1.6 to <2.0 m^2^; XL group, extra-large dialyzer surface area, ≥2.0 m^2^. ^a^Adjusted for age, sex, duration of dialysis, presence or absence of diabetes, and cardiovascular disease. ^b^Adjusted for clinicodemographic factors and dialysis-related factors, including Kt/V, β_2_-microglobulin level, ultrafiltration rate, dialysis time, and type of dialyzer. ^c^Adjusted for clinicodemographic and dialysis-related factors, C-reactive protein, hemoglobin, normalized protein catabolic rate, serum albumin, body mass index, and simplified creatinine index. CI, confidence interval; HR, hazard ratio.
