# Supplementary material for: Dialyzer surface area is a significant predictor of mortality in patients on hemodialysis: a 3-year nationwide cohort study
Source: Sci Rep. 2021 Oct 18;11:20616. doi: 10.1038/s41598-021-99834-4 (PMC8523692; doi:10.1038/s41598-021-99834-4)
Supplement: Supplementary file 10 — Supplementary Table S7. [file 41598_2021_99834_MOESM10_ESM.docx]

**Supplementary Table 7.** Comparison of hazard ratios (95% confidence intervals) for all-cause mortality according to dialyzer surface area and when stratified by median values for age, body mass index, β2-microglobulin and serum albumin levels, and comorbid cardiovascular disease or diabetes mellitus at baseline

| Group | Age <68 years | | |  | Age ≥68 years | | |
| --- | --- | --- | --- | --- | --- | --- | --- |
|  | HR | 95% CI | P-value |  | HR | 95% CI | P-value |
| S | 1.12 | 1.04–1.20 | 0.001 |  | 1.14 | 1.10–1.17 | < 0.0001 |
| M | 1.00 | Reference | - |  | 1.00 | Reference | - |
| L | 0.95 | 0.89–1.02 | 0.190 |  | 0.91 | 0.87–0.94 | < 0.0001 |
| XL | 0.88 | 0.82–0.94 | 0.0003 |  | 0.79 | 0.75–0.83 | < 0.0001 |
|  |  |  |  |  |  |  |  |
| Group | CVD | | |  | No CVD | | |
|  | HR | 95% CI | P-value |  | HR | 95% CI | P-value |
| S | 1.06 | 1.01–1.11 | 0.018 |  | 1.05 | 1.01–1.09 | 0.011 |
| M | 1.00 | Reference | - |  | 1.00 | Reference | - |
| L | 0.92 | 0.88–0.96 | 0.0004 |  | 0.97 | 0.93–1.01 | 0.152 |
| XL | 0.87 | 0.83–0.92 | < 0.0001 |  | 0.87 | 0.82–0.91 | < 0.0001 |
|  |  |  |  |  |  |  |  |
| Group | DM | | |  | No DM | | |
|  | HR | 95% CI | P-value |  | HR | 95% CI | P-value |
| S | 1.11 | 1.05–1.15 | < 0.0001 |  | 1.13 | 1.09–1.17 | < 0.0001 |
| M | 1.00 | Reference | - |  | 1.00 | Reference | - |
| L | 0.93 | 0.89–0.98 | 0.005 |  | 0.9 | 0.86–0.94 | < 0.0001 |
| XL | 0.86 | 0.81–0.91 | < 0.0001 |  | 0.78 | 0.74–0.82 | < 0.0001 |
|  |  |  |  |  |  |  |  |
| Group | BMI <21 | | |  | BMI ≥21 | | |
|  | HR | 95% CI | P-value |  | HR | 95% CI | P-value |
| S | 1.12 | 1.07–1.15 | < 0.0001 |  | 1.07 | 1.01–1.14 | 0.019 |
| M | 1.00 | Reference | - |  | 1.00 | Reference | - |
| L | 0.91 | 0.87–0.94 | < 0.0001 |  | 0.97 | 0.92–1.02 | 0.277 |
| XL | 0.8 | 0.75–0.85 | < 0.0001 |  | 0.89 | 0.83–0.94 | 0.0002 |
|  |  |  |  |  |  |  |  |
| Group | β2MG < 27 mg/L | | |  | β2MG ≥27 mg/L | | |
|  | HR | 95% CI | P-value |  | HR | 95% CI | P-value |
| S | 1.06 | 1.01–1.11 | 0.008 |  | 1.07 | 1.03–1.10 | 0.0003 |
| M | 1.00 | Reference | - |  | 1.00 | Reference | - |
| L | 0.94 | 0.89–0.98 | 0.015 |  | 0.93 | 0.90–0.97 | 0.0007 |
| XL | 0.87 | 0.82–0.93 | < 0.0001 |  | 0.86 | 0.82–0.89 | < 0.0001 |
|  |  |  |  |  |  |  |  |
| Group | Albumin <3.7 g/dL | | |  | Albumin ≥3.7 g/dL | | |
|  | HR | 95% CI | P-value |  | HR | 95% CI | P-value |
| S | 1.08 | 1.05–1.11 | < 0.0001 |  | 1.07 | 1.00–1.13 | 0.037 |
| M | 1.00 | Reference | - |  | 1.00 | Reference | - |
| L | 0.94 | 0.90–0.97 | 0.0013 |  | 0.96 | 0.90–1.01 | 0.169 |
| XL | 0.86 | 0.81–0.90 | < 0.0001 |  | 0.87 | 0.82–0.94 | 0.0002 |

S group, small dialyzer surface area, <1.5 m^2^; M group, medium dialyzer surface area, 1.5 m^2^; L group, large dialyzer surface area, 1.6 to <2.0 m^2^; XL group, extra-large dialyzer surface area, ≥2.0 m^2^. β2MG, β_2_-microglobulin; BMI, body mass index; CI, confidence interval; CVD, cardiovascular disease; HR, hazard ratio.
