# Supplementary material for: Dialyzer surface area is a significant predictor of mortality in patients on hemodialysis: a 3-year nationwide cohort study
Source: Sci Rep. 2021 Oct 18;11:20616. doi: 10.1038/s41598-021-99834-4 (PMC8523692; doi:10.1038/s41598-021-99834-4)
Supplement: Supplementary file 11 — Supplementary Table S8. [file 41598_2021_99834_MOESM11_ESM.docx]

**Supplementary Table 8.** Hazard ratios (with 95% confidence intervals) for all-cause mortality according to dialyzer surface area and all-cause mortality stratified by Kt/V quartile

| Group | Kt/V <1.26 | | |  | Kt/V 1.26–1.40 | | |  | Kt/V 1.41–1.58 | | |  | Kt/V ≥1.59 | | |
| --- | --- | --- | --- | --- | --- | --- | --- | --- | --- | --- | --- | --- | --- | --- | --- |
|  | HR | 95% CI | P-value |  | HR | 95% CI | P-value |  | HR | 95% CI | P-value |  | HR | 95% CI | P-value |
| S | 1.11 | 1.06–1.17 | < 0.0001 |  | 1.05 | 0.98–1.12 | 0.167 |  | 1.01 | 0.95–1.08 | 0.701 |  | 1.13 | 1.06–1.20 | 0.0002 |
| M | 1.00 | Reference | - |  | 1.00 | Reference | - |  | 1.00 | Reference | - |  | 1.00 | Reference | - |
| L | 0.93 | 0.88–0.99 | 0.023 |  | 0.97 | 0.91–1.04 | 0.501 |  | 0.91 | 0.84–0.97 | 0.004 |  | 0.92 | 0.86–0.98 | 0.017 |
| XL | 0.86 | 0.80–0.93 | 0.0002 |  | 0.85 | 0.78–0.91 | < 0.0001 |  | 0.81 | 0.74–0.88 | < 0.0001 |  | 0.81 | 0.75–0.89 | < 0.0001 |

S group, small dialyzer surface area, <1.5 m^2^; M group, medium dialyzer surface area, 1.5 m^2^; L group, large dialyzer surface area, 1.6 to <2.0 m^2^; XL group, extra-large dialyzer surface area, ≥2.0 m^2^. CI, confidence interval; HR, hazard ratio.
